# Tubulointerstitial inflammation and fibrosis induced by proteinuria: fresh insights

**DOI:** 10.3389/fphar.2026.1837046

**Published:** 2026-05-28

**Authors:** Monica Cortinovis, Giuseppe Remuzzi, Norberto Perico

**Affiliations:** Istituto di Ricerche Farmacologiche Mario Negri IRCCS, Bergamo, Italy

**Keywords:** albuminuria, chronic kidney disease, inflammation, kidney fibrosis, proteinuria, tubular injury, tubular toxicity

## Abstract

Proteinuria is a well-established risk factor for chronic kidney disease progression, and its reduction is associated with improved kidney outcomes. During proteinuric chronic kidney diseases, the abnormal passage of plasma-derived proteins through the damaged glomerular filtration barrier can lead to the activation of various pathophysiological processes in the tubular compartment. These mainly comprise complement activation and excessive proximal tubule reuptake of filtered proteins, such as albumin and associated lipids, which trigger cytotoxic effects through the dysregulation of multiple signaling pathways. Collectively, these signaling events can induce proximal tubular cell apoptosis or the acquisition of injury phenotypes, characterized by the paracrine release of bioactive substances that promote infiltration of immune cells and fibroblast-to-myofibroblast differentiation, eventually contributing to the development of tubulointerstitial fibrosis and progressive loss of kidney function. In this review, we provided an up-to-date overview of the complex mechanisms behind the toxic effects of ultrafiltered proteins on the tubulointerstitium under proteinuric conditions. The mechanistic insights that have been gained recently could be valuable for the future development of new classes of drugs to be used in combination with, or as an alternative to, the currently approved anti-proteinuric treatments in patients who are poorly responsive or unable to tolerate them, respectively.

## 1 Introduction

Proteinuria is defined as the abnormal presence of proteins in the urine, including both albumin and other proteins. It varies in magnitude and can be either transient or persistent. Transient proteinuria can occur under different circumstances, such as during heavy physical exertion, fever, or urinary tract infections, and has a benign clinical course (Haider and Aslam, 2026). Persistently increased urinary excretion of total proteins or albumin, however, is a marker of kidney damage. Nowadays, assessment of albuminuria, together with estimated glomerular filtration rate, serves as the basis for chronic kidney disease diagnosis and staging according to the Kidney Disease: Improving Global Outcomes (KDIGO) clinical practice guidelines (KDIGO, 2024). Although other markers of kidney damage/function may also be used, based on KDIGO guidelines, to diagnose chronic kidney disease, this is generally defined by urinary albumin-to-creatinine ratio (ACR) ≥ 30 mg/g (approximately equivalent to urinary protein-to-creatinine ratio [PCR] ≥ 150 mg/g) and/or estimated glomerular filtration rate <60 mL/min/1.73 m^2^ for 3 months or longer (KDIGO, 2024). Since the severity of urinary albumin or total protein excretion has been associated with an increased risk of chronic kidney disease progression and mortality, three categories of persistent albuminuria have been established: A1, normal or mild increase (urinary ACR <30 mg/g, approximately equivalent to urinary PCR <150 mg/g); A2, moderate increase (urinary ACR 30–300 mg/g, approximately equivalent to urinary PCR 150–499 mg/g); A3, severe increase (urinary ACR ≥300 mg/g, approximately equivalent to urinary PCR ≥500 mg/g) (KDIGO, 2024). Moreover, the presence of nephrotic-range proteinuria (≥3.5 g per 24 h) and hypoalbuminemia define nephrotic syndrome, which is often but not necessarily accompanied by edema and dyslipidemia (KDIGO, 2021). Most forms of persistent proteinuria (and albuminuria) are caused by damage to the filtration barrier, which can be the result of primary glomerular diseases (e.g., minimal change disease, focal and segmental glomerulosclerosis, membranous nephropathy, IgA nephropathy) or glomerulopathies secondary to systemic diseases (e.g., diabetic kidney disease, lupus nephritis, antineutrophil cytoplasmic antibody-associated vasculitis). In patients with chronic glomerular diseases, pharmacological interventions that reduce urinary protein excretion, such as renin-angiotensin-aldosterone blockers and sodium-glucose co-transporter 2 (SGLT-2) inhibitors, slow kidney function decline (GISEN, 1997; Brenner et al., 2001; Perkovic et al., 2019; Heerspink et al., 2020; The EMPA-KIDNEY Collaborative Group et al., 2023; Cortinovis et al., 2025). Studies in experimental models of proteinuric chronic kidney diseases showed that chronic treatment with renin-angiotensin-aldosterone system blockers reduced proteinuria by ameliorating the glomerular sieving function (Remuzzi et al., 1990, 1993) and prevented progression or even promoted regression of glomerulosclerotic, vascular, and tubulointerstitial lesions (Remuzzi et al., 2006; Ruggenenti et al., 2012). These observations, along with a wealth of evidence from *in vitro* and *in vivo* studies documenting the toxic effects of filtered proteins on the tubulointerstitium, suggest that proteinuria may contribute directly to disease progression (Abbate et al., 2006). Under healthy conditions, only a tiny fraction of large proteins, such as albumin, evade the glomerular filtration barrier, and these are almost completely reabsorbed in the proximal tubule via clathrin-dependent endocytosis mediated by the megalin-cubilin receptor complex (Devuyst and Ronco, 2023; Hall, 2025). After cell internalization, proteins are released from their receptor within acidified endosomes and trafficked to lysosomes for degradation into amino acids, which return to the bloodstream via the basolateral membrane (Devuyst and Ronco, 2023; Hall, 2025). An alternative pathway to albumin degradation involves its dissociation from the megalin-cubilin complex within endosomes, followed by binding to the neonatal Fc receptor (FcRn), which mediates transcytosis of the intact protein into the circulation (Molitoris and Wagner, 2023). In the setting of glomerular diseases, increased amounts of proteins pass through the damaged filtration barrier and are retrieved in the proximal tubule, resulting in the activation of multiple pathophysiological processes. These events can cause proximal tubular cell apoptosis or the acquisition of phenotypic changes characterized by the paracrine release of bioactive factors that promote inflammatory cell recruitment and myofibroblast differentiation, ultimately contributing to the development of tubulointerstitial fibrosis and progressive kidney function decline (Zoja et al., 2015; Cortinovis et al., 2024). Indeed, in patients with proteinuric glomerulopathies, the degree of interstitial fibrosis on kidney biopsies is a strong predictor of disease progression (Mariani et al., 2018). In addition to reflecting glomerular diseases, persistent proteinuria may also result from impaired tubular protein handling. Tubular proteinuria is typically characterized by increased urinary excretion of low-molecular-weight proteins, such as β2-microglobulin and retinol binding protein 1. Examples include inherited disorders due to pathogenic variants in genes encoding for megalin (*LRP2*) or cubilin (*CUBN*). Specifically, *LRP2* variants lead to Donnai-Barrow syndrome, a rare autosomal recessive disorder characterized by facial dysmorphia, ocular problems, sensorineural hearing loss, low-molecular-weight proteinuria and, in most patients, normal kidney function, although cases of progressive estimated glomerular filtration rate decline and focal segmental glomerulosclerosis have been reported (Nielsen et al., 2016). Variants in *CUBN* have been shown to cause Imerslund-Gräsbeck syndrome, another autosomal recessive disorder characterized by intestinal malabsorption of vitamin B12, resulting in megaloblastic anemia, and low-molecular-weight proteinuria (Kingma et al., 2023). Imerslund-Gräsbeck syndrome-related variants were located in the N-terminal of *CUBN* or within the vitamin B12 binding domain. More recently, biallelic *CUBN* variants clustered in the C-terminal of the protein have been identified as a cause of isolated albumin-predominant proteinuria without kidney dysfunction (Ovunc et al., 2011; Bedin et al., 2020; Domingo-Gallego et al., 2022). The benign nature of chronic proteinuria associated with C-terminal *CUBN* variants can be explained by the reduced reabsorption of filtered proteins–especially albumin–by proximal tubular cells, and the consequent cytotoxicity (Simons, 2018). In this review, we provide an up-to-date overview of the complex mechanisms through which the excessive protein leakage across the disrupted glomerular filtration barrier can promote cytotoxicity, tubulointerstitial inflammation and kidney fibrosis.

## 2 Glomerulus-to-tubule signaling

**FIGURE 1:**
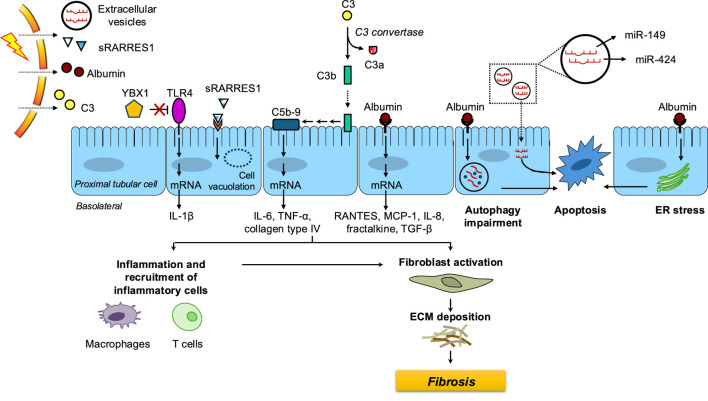
*Overview of the mechanisms underlying the development of tubulointerstitial inflammation and fibrosis in proteinuric chronic kidney diseases*. In proteinuric conditions, injurious factors released from damaged podocytes (e.g., extracellular vesicle-enclosed microRNAs and soluble retinoic acid receptor responder protein-1), along with the reduced secretion of protective factors (e.g., Y-box binding protein 1), and abnormally filtered plasma proteins, such as albumin and complement components, can lead to the activation of pathophysiological processes in the tubular compartment. These events can induce proximal tubular cell apoptosis–through mechanisms that can entail endoplasmic reticulum stress or autophagy dysfunction–or the acquisition of injury phenotypes, characterized by the paracrine release of bioactive mediators that promote inflammatory cell infiltration and fibroblast activation, eventually contributing to the development of tubulointerstitial fibrosis. Abbreviations: ECM, extracellular matrix; ER, endoplasmic reticulum; IL-1β, interleukin-1β; IL-6, interleukin-6; IL-8, interleukin-8; MCP-1, monocyte chemoattractant protein-1; miR-149, miRNA 149; miR-424, miRNA 424; RANTES, regulated upon activation, normal T-cell expressed and secreted; sRARRES1, soluble retinoic acid receptor responder protein-1; TGF-β, transforming growth factor-β; TLR4, Toll-like receptor-4; TNF-α, tumor necrosis factor-α; uPA, urokinase-type plasminogen activator; YBX1, Y-box binding protein 1.

During proteinuric chronic kidney diseases, injurious factors released from damaged glomerular cells, coupled with the reduced secretion of protective factors, and aberrantly filtered plasma components such as albumin and associated free fatty acids or complement factors, can lead to the activation of pathophysiological processes in the tubular compartment (Figure 1).

### 2.1 Podocyte-derived toxic and protective factors

The glomerular filtration barrier is composed of three layers that are critical for the ultrafiltration process: the fenestrated endothelium, the glomerular basement membrane and podocytes. Damage to any of these layers can lead to proteinuria. Among the several factors that can contribute to podocyte injury, increasing research interest is focused on indoxyl sulfate (Ichii et al., 2014; Jia et al., 2024), a uremic toxin which accumulates in the plasma of patients with advanced stages of chronic kidney disease, largely bound to albumin (Wu et al., 2011). Chronic administration of indoxyl sulfate to healthy mice led to podocyte foot process effacement, along with reduced expression and altered staining pattern for synaptopodin and podocin (Ichii et al., 2014). Further *in vitro* studies in immortalized mouse and human podocytes revealed that indoxyl sulfate caused activation and nuclear translocation of aryl-hydrocarbon receptor, resulting in lowered expression of podocyte-specific genes and increased transcription of pro-inflammatory mediators, eventually reducing cell viability (Ichii et al., 2014). Whether podocyte injury is mediated by either free or albumin-bound indoxyl sulfate is a matter of ongoing investigation. Among the three layers of the glomerular filtration barrier, to date only damage to podocytes has been associated with downstream tubular damage, through a variety of mechanisms. Following injury, podocyte transmembrane proteins can be cleaved into soluble forms, which participate in tubular damage. An example of such proteins is retinoic acid receptor responder protein-1 (RARRES1), whose expression is upregulated in kidney biopsies of patients with glomerular diseases (Chen et al., 2020). In murine models of diabetic kidney disease and focal segmental glomerulosclerosis, podocyte-specific overexpression of RARRES1, but not of the cleavage mutant, worsened glomerular and tubular injuries, pointing to soluble RARRES1 as the major culprit of the observed effects (Feng et al., 2024). Podocyte-derived RARRES1 was taken up by proximal tubular cells *in vivo*, leading to tubular injury characterized by tubular vacuolation and lipid accumulation (Feng et al., 2024). Although in the kidney RARRES1 is mostly expressed in podocytes, it was also found to be upregulated in glomerular endothelial cells in renal biopsies from patients with glomerular diseases (Möller-Hackbarth et al., 2021). In a mouse model of glomerulonephritis, overexpression and inactivation of RARRES1, specifically in endothelial cells, worsened and ameliorated podocyte injury, respectively (Möller-Hackbarth et al., 2021). Whether soluble RARRES1 can be released from glomerular endothelial cells and promote tubular injury remains to be investigated. Podocytes can also contribute to tubular damage through the secretion of specific extracellular vesicle (EVs)-enclosed microRNAs (miRNAs). For instance, EVs released from podocytes cultured under high glucose conditions, which mimic the environment of diabetic kidney disease, exhibited increased levels of miR-221 (Su et al., 2020). In transwell experiments, this miRNA was transferred into tubular epithelial cells, where it downregulated Dickkopf-related protein 2 (DKK2), a suppressor of Wnt/β-catenin signaling, leading to the acquisition of a dedifferentiation state that is involved in tubulointerstitial fibrosis (Su et al., 2020). In line with this, EVs isolated from puromycin-treated podocytes were found to induce apoptosis of cultured tubular epithelial cells *via* activation of the p38/extracellular signal-regulated kinase (ERK) pathway, an effect that was at least partially mediated by the upregulation and transfer of miR-149 and miR-424 (Jeon et al., 2020). In addition to the release of injurious factors, the loss of podocyte-derived protective factors can also promote tubulointerstitial injury. This is the case for the cold-shock protein Y-box binding protein 1 (YBX1), which under physiological conditions is constitutively secreted by podocytes and inhibits the Toll-like receptor-4 (TLR4)-NF-κB signaling in tubular epithelial cells, thereby preventing spontaneous and sterile inflammation (Rana et al., 2024). Following podocyte injury, reduced release of this protective factor renders tubular epithelial cells more susceptible to an inflammatory milieu, as evidenced by the finding that mice expressing a secretion-deficient YBX1 protein specifically in podocytes developed more severe tubular injury induced by the TLR4 ligand lipopolysaccharide than their wild-type counterparts (Rana et al., 2024).

### 2.2 Tubular complement activation in proteinuric chronic kidney diseases

**FIGURE 2:**
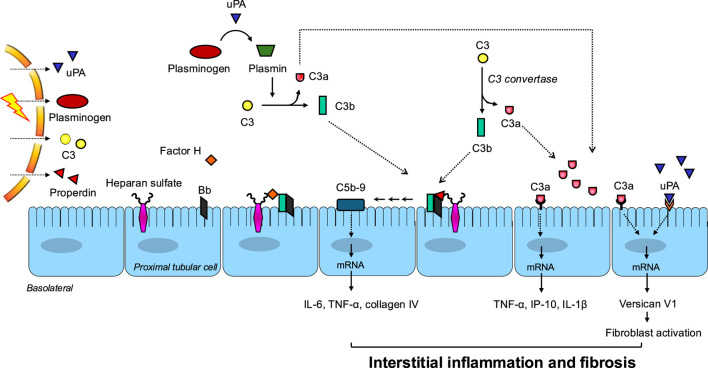
*Complement activation on the tubular brush border and its consequences in proteinuric states*. During proteinuric conditions, aberrantly filtered complement components are activated in the tubular compartment via the alternative complement pathway, which is initiated by properdin binding to heparan sulfate. Moreover, active plasmin produced by uPA-mediated cleavage of ultrafiltered plasminogen takes part in the formation of anaphylatoxins through a non-canonical pathway. Upon complement activation, C3a binds to its receptor at the apical side of the tubule, and upregulates the expression of pro-inflammatory cytokines (TNF-α, IP-10 and IL-1β) and, by acting in synergy with uPA, induces the expression of versican V1. In addition to C3a, C5b-9 stimulates the production of pro-inflammatory (IL-6, TNF-α) and pro-fibrotic (collagen type IV) factors in proximal tubular epithelial cells. As a consequence, tubulointerstitial inflammation and fibrosis occurs. Abbreviations: Bb, fragment of complement factor B produced by activation of the alternative pathway; IL-1β, interleukin-1β; IL-6, interleukin-6; IP-10, interferon gamma-induced protein-10; TNF-α, tumor necrosis factor-α; uPA, urokinase-type plasminogen activator.

Tubular complement activation occurs in patients with proteinuric chronic kidney diseases, as evidenced by tubular deposition of C3d and the membrane attack complex C5b-9 in their kidney biopsies (Koopman et al., 2020; Alkaff et al., 2024, 2025). The currently available evidence suggests that the alternative pathway plays a major role in tubular complement activation under proteinuric conditions (Figure 2). Indeed, kidney biopsies from patients with various proteinuric kidney diseases showed that properdin (alternative pathway), but not C1q (classical pathway) or mannose-binding lectin-associated serine protease 2 (lectin pathway), consistently exhibited overlapping distribution with C5b-9 deposition at the apical surface of the tubules (Alkaff et al., 2025). Moreover, *in vitro* experiments revealed that properdin, which stabilizes the alternative pathway C3 convertase, could bind to the brush border of proximal tubular epithelial cells in a dose-dependent manner, and control complement activation (Gaarkeuken et al., 2008). Properdin binding to tubular cells appears to be dependent on heparan sulfate proteoglycans, since it was prevented by pre-treatment of the cells with heparitinase (Zaferani et al., 2011; Lammerts et al., 2020). In addition, properdin colocalized with the heparan sulfate proteoglycan syndecan-1 at the apical side of the tubules in kidney biopsies of patients with proteinuric kidney diseases (Alkaff et al., 2025). Factor H, the main soluble inhibitor of the alternative complement pathway, can also bind to tubular heparan sulfates, but to a different epitope than that recognized by properdin (Zaferani et al., 2012). The addition of albumin to cultured proximal tubular epithelial cells reduced heparan sulfate density on their surface, as well as binding to exogenous factor H, suggesting that proteinuria may lower the ability of these cells to counteract local complement activation (Buelli et al., 2009).

Proximal tubular epithelial cells are able to synthesize C3 and other complement components (Pratt et al., 2002), and to upregulate C3 in response to serum proteins *in vitro* (Tang et al., 2001). The relative contribution of plasma-derived C3 and tubular cell-derived C3 in proteinuria-induced tubulointerstitial injury has been studied in experiments involving the transplantation of C3-deficent kidneys into wild-type mice or vice versa in a mouse model of protein-overload proteinuria (Abbate et al., 2008). Protein overload in wild-type mice transplanted with C3-deficent kidneys led to glomerular injury, C3 accumulation in proximal tubules, and interstitial infiltration of macrophages. On the other hand, protein overload in C3-deficient mice transplanted with wild-type kidneys resulted in milder disease without abnormal C3 staining (Abbate et al., 2008). These findings suggest that ultrafiltered C3 contributes more significantly than locally synthesized C3 to tubulointerstitial damage induced by proteinuria (Abbate et al., 2008).

Regardless of the source of complement factors, intratubular complement activation eventually leads to cytotoxic, inflammatory and fibrogenic effects in the tubulointerstitial compartment (Abbate et al., 2006). For instance, the insertion of C5b-9 into the plasma membrane of proximal tubular epithelial cells resulted in the secretion of inflammatory cytokines, such as IL-6 and TNF-α, *in vitro* (David et al., 1997) and in the upregulation of collagen type IV gene expression in mice with adriamycin-induced nephropathy, a commonly used model of proteinuric chronic kidney disease (Abe et al., 2004). In the same animal model, C3a and soluble urokinase-type plasminogen activator (uPA) receptor were found to synergistically induce tubular cell expression of the extracellular matrix protein versican V1, which in turn promoted interstitial fibrosis by activating the CD44/Smad3 pathway in fibroblasts (Han et al., 2019). Upon binding to its receptor on tubular epithelial cells, C3a can also contribute to tubulointerstitial fibrosis by acting in concert with TGF-β to facilitate the assembly and activation of the NLRP3 (NLR Family Pyrin Domain Containing 3) inflammasome, as observed *in vitro* and in mice with unilateral ureteral obstruction (You et al., 2022, 2023). The hypothesis that complement plays a pathogenic role in tubulointerstitial disease is also supported by studies that show that, in animal models of proteinuria, complement depletion (Morita et al., 1997; Nomura et al., 1997), C6-deficiency (Nangaku et al., 1999) or complement inhibitor molecules (Nomura et al., 1997; He et al., 2005) reduced tubulointerstitial injury.

Under proteinuric conditions, aberrantly filtered uPA and plasminogen can promote complement activation in the tubular lumen through a non-canonical pathway. Notably, endogenous uPA in urine from healthy individuals was sufficient to activate complement, with the formation of C3a and C5a anaphylatoxins when the exogenous plasminogen and purified complement components C3 and C5 were added (Isaksson et al., 2024). These reactions were inhibited by the potassium-sparing diuretic amiloride, an off-target uPA antagonist. Moreover, in mice with inducible podocin knockout–an animal model of massive proteinuria–treatment with an anti-uPA monoclonal antibody reduced urinary anaphylatoxin excretion and kidney tissue expression of the NLRP3 inflammasome protein, without affecting proteinuria (Isaksson et al., 2024). Similarly, in mice with chronic hypertension and albuminuria caused by reduced kidney mass combined with mineralocorticoids and high dietary salt, deletion of the uPA gene led to lower urinary excretion of C3a, along with reduced kidney levels of the pro-inflammatory cytokines interferon gamma-induced protein-10 and TNF, compared to levels observed in wild-type littermates (Bach et al., 2026). Together, these findings suggest that during proteinuria, active plasmin generated by uPA-mediated cleavage of plasminogen participates in the formation of anaphylatoxins and downstream pro-inflammatory signaling in the tubular lumen (Figure 2).

## 3 Tubular injury pathways linked to proteinuria

**FIGURE 3:**
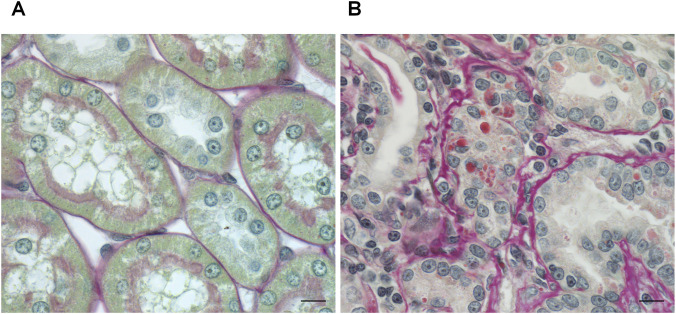
*Excessive tubular reuptake of ultrafiltered proteins in proteinuric conditions.* PAS stained representative histological sections of the renal cortex of a normal kidney **(A)** and of a remnant kidney 6 months after 5/6 renal mass ablation **(B)** in Sprague Dawley rats. In contrast to tubular cells of the normal rat kidney, those of the remnant kidney showed prominent cytoplasmic droplets, corresponding to endocytic vesicles containing reabsorbed proteins. The scale bar in the images is 10 μm.

Excessive tubular reuptake of ultrafiltered proteins can lead to the activation of apoptotic pathways or adaptive responses, such as endoplasmic reticulum stress or autophagy, which are initially protective, but may eventually end in tubular cell injury or death in cases of severe or prolonged stress (Figure 3).

### 3.1 Apoptosis and other types of programmed cell death

Apoptosis of renal tubular cells has been observed following cell culture stimulation with albumin, in animal models of proteinuria and in patients with nephrotic syndrome (Erkan et al., 2001, 2005; Li et al., 2010; Eleftheriadis et al., 2023). Both the extrinsic (Erkan et al., 2001; Eleftheriadis et al., 2023) and intrinsic (Erkan et al., 2007) pathways have been shown to contribute to albumin-induced apoptosis of proximal tubular cells *in vitro*. The involvement of the extrinsic pathway, which begins with a pro-death signal originating from outside the cell, was suggested by caspase-8 activation, both in porcine and human proximal tubular cells after albumin overload (Erkan et al., 2001; Eleftheriadis et al., 2023). The downregulation of Akt, a protein kinase B that regulates albumin endocytosis in the proximal tubule (Koral et al., 2014), has recently been linked to proximal tubular epithelial cell apoptosis via the activation of the intrinsic mitochondrial pathway under proteinuric conditions (Chhaing et al., 2025). Specifically, the chemical inhibition and overexpression of Akt exacerbated and mitigated apoptosis, respectively, in cultured proximal tubular cells in response to albumin exposure. Moreover, in mice with specific Akt1 and Akt2 knockout in proximal tubule cells, albumin overload led to the dephosphorylation and translocation of the downstream Akt target Forkhead box O-1 to the nucleus, where it stimulated gene expression of the pro-apoptotic B cell lymphoma (Bcl)-2 family member BIM (Chhaing et al., 2025). This was followed by the passage of BIM and Bax, another pro-apoptotic protein, to the mitochondrial membrane and cytochrome c release to the cytoplasm, causing apoptosis *via* caspase-9 activation (Erkan et al., 2007; Chhaing et al., 2025). Notably, reduced expression of phosphorylated Akt, the active form of the kinase, was observed in proximal tubular epithelial cells of patients with focal and segmental glomerulosclerosis early in the disease course, which preceded the development of kidney failure (Chhaing et al., 2025). Collectively, these findings suggest associations between reduced Akt activity in the proximal tubule, cell apoptosis and kidney disease progression.

In the setting of chronic kidney disease, increased levels of oxidative stress and uremic retention of solutes–such as advanced glycation end-products and urea–enhance post-translational modifications of albumin, including oxidation, glycation and carbamylation (Noels et al., 2024). These post-translational changes of albumin alter its proximal tubule handling. Specifically, they reduce albumin binding to the FcRn receptor within the endosomal compartment, thereby lowering transtubular retrieval of the intact protein, which is instead trafficked to the lysosomes for degradation (Molitoris et al., 2022). This intracellular molecular sorting pathway enables the return of physiological albumin to the circulation and the catabolism of potentially toxic forms of the protein. In chronic kidney disease, increased oxidation, glycation and carbamylation of albumin could lead to lysosome overloading, which might result in proximal tubule injury (Molitoris et al., 2022). In fact, these modified forms of albumin appear to be more cytotoxic to tubular cells than the native protein (Gross et al., 2011; Qi et al., 2015; Zhang et al., 2025). For instance, in cultured tubular epithelial cells, oxidized albumin–but not unmodified albumin–triggered ferroptosis, a form of regulated cell death characterized by iron-dependent lipid peroxidation and intracellular reactive oxygen species (ROS) accumulation (Zhang et al., 2025). Consistent with this, intraperitoneal injection of oxidized albumin in mice with adriamycin-induced nephropathy exacerbated glomerular and tubular injury through mechanisms involving the impairment of antioxidant defenses and activation of ferroptotic cell death (Zhang et al., 2025). The induction of oxidative stress also contributed to the glycated albumin-mediated apoptosis of tubular epithelial cells (Qi et al., 2015). The observation that elevated circulating levels of glycated or carbamylated albumin in patients with chronic kidney disease have been associated with greater risks of progression to kidney failure and mortality suggests that albumin derivatives may play a pathogenic role (Kalim et al., 2023; Tang et al., 2024; Folttmann et al., 2025).

In addition to circulating proteins, free fatty acids (FFAs) noncovalently bound to albumin can also pass into the ultrafiltrate when there is glomerular filtration barrier disruption. Within the luminal space, FFAs dissociate from albumin (Garcia et al., 2025), undergo proximal tubular reabsorption primarily through the apical fatty acid transport protein-2 (FATP2), and contribute to cytotoxicity by causing lipoapoptosis (Khan et al., 2018). Indeed, overload of lipidated albumin in mice with global deletion of the FATP2 gene (*Slc27a2*) resulted in lower proximal tubular epithelial cell apoptosis and tubular atrophy compared to what was observed in wild-type animals (Khan et al., 2018). Other receptors implicated in the proximal tubular cell uptake of FFAs include kidney injury molecule-1 (KIM-1) (Mori et al., 2021) and CD36 (Susztak et al., 2005), which are upregulated in patients with diabetic kidney disease (Mori et al., 2021) and nephrotic syndrome (Baines et al., 2012), respectively. In cultured proximal tubular cells, endocytosis of FFA-bound albumin mediated by either KIM-1 or CD36 led to cell death, apoptosis and inflammation (Li et al., 2018; Mori et al., 2021). Moreover, both *in vitro* and in mice on a high-fat diet, empagliflozin was reported to attenuate FFA-induced tubular cell apoptosis by down-regulating CD36 (Huang et al., 2021).

### 3.2 Endoplasmic reticulum stress

Excessive reabsorption of filtered proteins can also promote tubular cell injury by driving endoplasmic reticulum (ER) stress, a cellular response to the accumulation of misfolded proteins within the ER, which induces the activation of the unfolded protein response (Byun et al., 2025). This signaling cascade initially aims to remove improperly folded proteins and restore ER homeostasis, but it can be rewired to trigger apoptosis in case of prolonged or excessive stress (Almanza et al., 2019). Albumin overload in cultured tubular epithelial cells led to the activation of RNA-dependent protein kinase R (PKR)-like endoplasmic reticulum kinase (PERK), one of the ER transmembrane receptors, and the upregulation of the downstream transcription factor C/EBP homologous protein (CHOP), which promoted apoptosis (Wu et al., 2010). Consistent with this, lowering the increased CHOP expression in *db/db* diabetic mice using antisense oligonucleotides reduced apoptotic cell death both in the tubular and glomerular compartments, while also mitigating albuminuria and interstitial fibrosis (Shahzad et al., 2021). A study involving the use of two murine models of nephrotic syndrome identified thioredoxin-interacting protein (TXNIP) – a redox protein that binds to and inhibits the antioxidant enzyme thioredoxin (Trx) – as a key signaling node that links ER stress with apoptosis and inflammation under proteinuric conditions (Park et al., 2022). In particular, in response to albuminuria-induced ER stress in tubular epithelial cells, CHOP promotes TXNIP translocation from the nucleus to mitochondria, where it is required for ROS generation (Park et al., 2022). The increased mitochondrial ROS then leads to Trx2 oxidation, which dissociates from the apoptosis signal regulating kinase 1 (ASK1), allowing it to initiate mitochondria-dependent apoptosis (Park et al., 2022). Oxidized Trx2 also releases TXNIP, which then associates with NLRP3 and activates inflammasome. The involvement of ER stress in albumin overload-induced inflammasome activation in tubular cells has also been observed in a mouse model of diabetic nephropathy (Fang et al., 2013).

Albumin overload downregulates the kidney-protective protein Klotho in tubular cells, at least in part by activating the ER stress pathway, as suggested by a study carried out in cultured kidney epithelial cells and in the POD-ATTACK (podocyte apoptosis through targeted activation of caspase-8) mouse model of proteinuric kidney disease (Delitsikou et al., 2020). The exposure of tubular epithelial cells to albumin activates the ER stress components activating transcription factor 3 (ATF3) and ATF4, which repress Klotho transcription, thereby reducing protein expression (Delitsikou et al., 2020). Inhibiting ER stress with the chemical chaperone sodium phenylbutyrate reversed the effect of albumin on Klotho protein levels *in vitro* and *in vivo* (Delitsikou et al., 2020).

### 3.3 Autophagy

Autophagy is a tightly regulated cellular process involving the lysosomal degradation of long-lived or aggregated proteins, damaged organelles and macromolecules, for clearance and reuse (Tang et al., 2020). The kidneys maintain high levels of baseline autophagy activity, especially in podocytes and tubular epithelial cells, to guarantee their homeostasis, structure and function (Bhatia and Choi, 2023). In the proximal tubule autophagy also promotes receptor-mediated transcytosis of albumin (Uchida et al., 2021).

**FIGURE 4:**
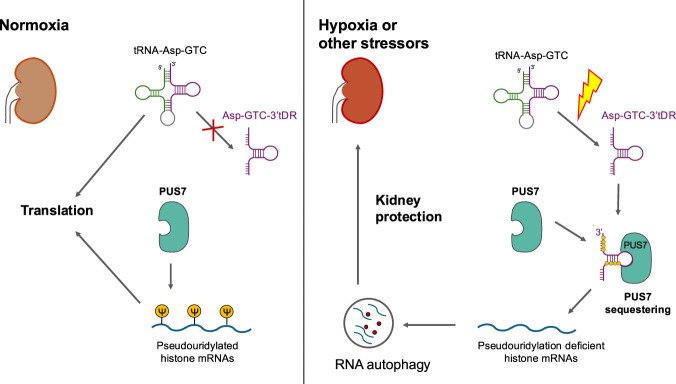
*tRNA-Asp-GTC-3′DR confers kidney protection by inducing RNA autophagy.* Under normoxic conditions, most mature tRNA-Asp-GTC in the kidneys remains uncleaved, and pseudouridine synthase 7 (PUS7) mediates pseudouridylation of target mRNAs, including histone mRNAs, enhancing their stability. In the kidney, hypoxia and other stress conditions promote tRNA-Asp-GTC cleavage, with the generation of two well-defined tRNA-derived RNAs (tDRs) from the 5′ end (tRNA-Asp-GTC-5′DR) and the 3′ end (tRNA-Asp-GTC-3′DR). By means of two internal 4-guanine motifs, tRNA-Asp-GTC-3′DR forms a peculiar structure and sequesters PUS7, thereby inhibiting pseudouridylation of nascent histone mRNAs, which are directed to autophagy. The upregulation of RNA autophagy provides kidney protection. Abbreviations: PUS7, pseudouridine synthase 7.

Following protein overload, the autophagic pathway is induced in the proximal tubule as an adaptive response against cellular injury. Indeed, the exposure of cultured proximal tubular epithelial cells to urinary proteins or serum albumin results in autophagy activation through a mechanism that involves oxidative stress (Liu et al., 2014). Moreover, both *in vitro* and *in vivo* studies have shown that increasing autophagy with the mammalian target of rapamycin (mTOR) inhibitor rapamycin reduced albumin-induced apoptosis of proximal tubular epithelial cells, whereas blocking autophagy with chloroquine had the opposite effect (Liu et al., 2014; Tan et al., 2018). However, excessive or prolonged albumin uptake can impair the autophagic flux in proximal tubular epithelial cells through an mTOR-dependent mechanism, thereby reducing the removal of long-lived proteins (Nolin et al., 2016). MiR-664a-5p also participates in autophagy dysfunction secondary to albumin overload in the proximal tubule (Shan et al., 2024). The expression of this miRNA was higher in the kidneys of mice with experimental membranous nephropathy and in cultured proximal tubular epithelial cells exposed to albumin (Shan et al., 2024). Mechanistic insights revealed that miR-664a-5p reduced the expression of the nuclear serine/threonine kinase homeodomain-interacting protein kinase 2, resulting in calpain 1/Gsα-mediated autophagy inhibition, which led to proximal tubular cell apoptosis (Shan et al., 2024). In addition to miRNA, other non-coding RNAs have also been implicated in the regulation of autophagy in the kidney under stress conditions. Of particular interest in this regard is one specific transfer RNA (tRNA)-derived fragment (tDRs) that originates from the 3′ end of tRNA-Asp-GTC, called tRNA-Asp-GTC-3′tDR, which was found to be upregulated in the kidneys in four experimental models of kidney injury: ischemia-reperfusion injury, unilateral ureteral obstruction, uninephrectomy, and albumin-induced protein overload (Li et al., 2025). In the former model, further elevating tRNA-Asp-GTC-3′tDR levels through synthetic tDR mimics delivered to the kidney enhanced autophagy while mitigating renal injury, inflammation and fibrosis. In contrast, the inhibition of this tDR using a specific antisense oligonucleotide had the opposite effect. Mechanistically, through the two internal 4-guanine motifs, tRNA-Asp-GTC-3′tDR adopts a peculiar structure which sequesters pseudouridine synthase 7, thereby preventing the pseudouridylation of histone mRNAs and directing them toward degradation by autophagy, a process termed RNA autophagy (Figure 4) (Li et al., 2025). Notably, tRNA-Asp-GTC-3′tDR levels were elevated in urine samples of pre-eclamptic patients with proteinuria and in the kidneys of patients with stage 3 chronic kidney disease, with the latter also exhibiting lower kidney expression of histone *H4C3* mRNA compared to patients with normal estimated glomerular filtration rate (Li et al., 2025). These findings underscore the therapeutic potential of future innovative strategies, such as synthetic tRNA-Asp-GTC-3′tDR mimics to augment kidney-specific RNA autophagy in the setting of kidney diseases (Table 1) (Bhatia and Choi, 2025). Potential therapeutic targets to protect proximal tubular cells from autophagy impairment caused by albumin, either alone or bound to free fatty acids, have also been identified in murine models of obesity-related nephropathy. Among these is the pathway involving the adipokine visceral adipose tissue-derived serine protease inhibitor (vaspin) and heat shock protein family A (Hsp70) member one like (HSPA1L). In this regard, mice knocked out for vaspin under a high-fat high-sucrose diet exhibited enlarged lysosomes and accumulation of the autophagy substrate p62 in proximal tubular cells (Nakatsuka et al., 2021). Further investigations revealed that vaspin forms a complex with HSPA1L and 78 kDa glucose-regulated protein (GRP78), which mediates vaspin uptake by proximal tubular cells and intracellular trafficking to lysosomes and endoplasmic reticulum, where it preserves organelle homeostasis and promotes autophagy (Nakatsuka et al., 2021). Albumin overload and reabsorption in proximal tubular cells leads to the secretion of HSPA1L, thereby reducing the amount of intracellular protein available for interaction with vaspin, ultimately resulting in autophagy impairment (Nakatsuka et al., 2021). Another study identified the transcription factor EB (TFEB), which is known to modulate the expression of genes involved in lysosomal exocytosis, as a protective factor against autophagy impairment induced by albumin enriched in fatty acids (Nakamura et al., 2023). Mice with proximal tubular epithelial cell-specific knockout for *Tfeb* on a high-fat diet exhibited enlarged lysosomes and autophagic stagnation, which made the kidneys more susceptible to injury following ischemia-reperfusion (Nakamura et al., 2023). Exposure to palmitic acid-bound albumin enhanced TFEB dephosphorylation and nucleus translocation in proximal tubular epithelial cells, which promoted lysosomal exocytosis at the apical membrane, thus alleviating autophagic stagnation (Nakamura et al., 2023). A further study in the high-fat diet model showed that empagliflozin reduced the diet-induced increase in albumin reabsorption mediated by megalin and the consequent autophagic demand in the proximal tubules, thereby preventing stagnation of autophagic flux (Matsui et al., 2025). Similar results were reported in mice with 5/6 nephrectomy (Matsui et al., 2025). These findings point to the restoration of autophagic activity in the proximal tubule as one of the mechanisms underlying the kidney-protective effects of SGLT-2 inhibitors.

**TABLE 1:** Novel potential therapeutic strategies to reduce proteinuria-induced tubulointerstitial injury explored in preclinical studies.

Therapeutic strategy	Target processes	*In vitro* findings	*In vivo* findings	Clinical relevance of the preclinical findings
tDR mimics (synthetic tDR mimics)	Autophagy	Transfection of synthetic tRNA-Asp-GTC-3′tDR mimics into cultured tubular epithelial cells induced autophagy	Kidney delivery of synthetic tRNA-Asp-GTC-3′tDR mimics in mice with renal ischemia/reperfusion injury increased autophagy and mitigated tubular injury, immune cell infiltration and fibrosis	Increased tRNA-Asp-GTC-3′tDR levels in urine samples of pre-eclamptic patients with proteinuria and in kidneys of patients with stage 3 chronic kidney disease
NNMT inhibition (5-amino-1-methylquinolinium)	Cellular senescence and kidney fibrosis	Small-molecule NNMT inhibitor reduced gene and protein expression of markers of senescence and fibrosis in TGF-β-treated cultured tubular epithelial cells	Small-molecule NNMT inhibitor treatment (SC) attenuated tubular injury, interstitial fibrosis and macrophage infiltration in mice with UUO	In kidney biopsies of patients with diabetic kidney disease, NNMT protein levels associated with markers of senescence and fibrosis
Scavenging IsoLG (pentylpyridoxamine)	Inflammation	IsoLG scavenger reduced IsoLG-modified apoAI uptake by cultured tubular epithelial cells, along with gene expression of KIM-1 (*HAVCR1*) and *NLRP3*	IsoLG scavenger treatment (PO) reduced albuminuria, tubular injury and interstitial fibrosis in a mouse model of proteinuria induced by podocyte-specific injury	Increased urinary levels of IsoLG-modified apoAI in patients with proteinuric kidney diseases
IGFBP7 inhibition (levomefolic acid)	Kidney fibrosis	Levomefolic acid reduced protein expression of fibrosis markers in high glucose-treated cultured tubular cells	Levomefolic acid treatment (IP) reduced glomerular injury and interstitial fibrosis in murine models of early and advanced DKD	In patients with DKD, serum IGFBP7 levels associated positively with DKD progression and albuminuria
Corisin inhibition (anti-corisin mAb)	Kidney fibrosis	Corisin increased gene expression of senescence and fibrosis markers in cultured tubular epithelial cells. These effects were inhibited by anti-corisin mAb	Anti-corisin mAb treatment (IP) attenuated tubular injury, macrophage infiltration and fibrosis in diabetic TGF-β transgenic mice with kidney fibrosis	In patients with DKD, higher serum corisin levels associated with subsequent faster eGFR decline

Abbreviations: apoAI, apolipoprotein AI; DKD, diabetic kidney disease; eGFR, estimated glomerular filtration rate; IGFBP7, insulin-like growth factor binding-protein 7; IP, intraperitoneal; IsoLG, isolevuglandin; KIM-1, kidney injury molecule-1; METTL3, methyltransferase-like 3; mAb, monoclonal antibody; NLRP3, NLR, family pyrin domain containing 3; NNMT, nicotinamide-N-methyltransferase; PO, per os; SC, subcutaneous; tDR, tRNA-derived small RNA; TGF-β, transforming growth factor-β; UUO, unilateral ureteral obstruction.

## 4 Proteinuria-induced functional and phenotypic maladaptive changes along the nephron

### 4.1 Cellular senescence

**FIGURE 5:**
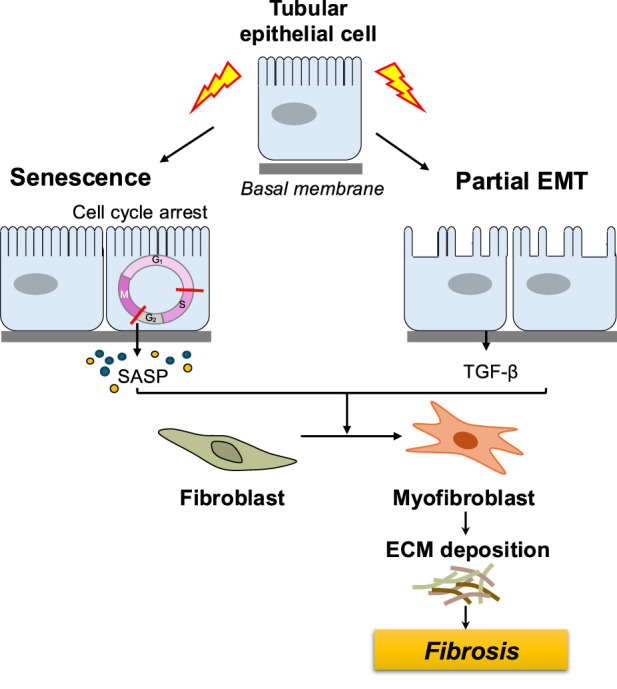
*Contribution of cellular senescence and partial epithelial-to-mesenchymal transition to the development of kidney fibrosis.* In response to injury, tubular epithelial cells can enter a state of senescence, characterized by irreversible cell-cycle arrest and the acquisition of a senescence-associated secretory phenotype, which includes pro-inflammatory and pro-fibrotic factors. The latter promote fibroblast-to-myofibroblast differentiation and extracellular matrix deposition, eventually contributing to the development of kidney fibrosis. Injured proximal tubular epithelial cells can also participate in kidney fibrosis through partial epithelial-to-mesenchymal transition. During this process, they remain attached to the basement membrane, while acquiring mesenchymal characteristics and the ability to produce cytokines and pro-fibrotic factors. Among these, transforming growth factor-β stimulates fibroblast-to-myofibroblast transformation and extracellular matrix accumulation. The senescence-associated secretory phenotype is rich in pro-fibrotic factors, including transforming growth factor-β, which could activate the epithelial-to-mesenchymal transition program in tubular epithelial cells. In turn, partial epithelial-to-mesenchymal transition can induce cell-cycle arrest of tubular epithelial cells at the G2/M phase. Thus, senescence and partial epithelial-to-mesenchymal transition of tubular epithelial cells appear to be intrinsically linked, even though a causal relationship between these two processes remains to be demonstrated. Abbreviations: ECM, extracellular matrix; EMT, epithelial-to-mesenchymal transition; SASP, senescence-associated secretory phenotype; TGF-β, transforming growth factor-β.

In response to an increased reabsorption workload, tubular epithelial cells can enter a state of senescence (Eleftheriadis et al., 2023), characterized by irreversible cell-cycle arrest and the acquisition of a distinct secretory phenotype consisting of various pro-inflammatory and pro-fibrotic molecules, which is known as senescence-associated secretory phenotype (Figure 5) (Chanvillard et al., 2025). In patients with proteinuric chronic kidney diseases–such as focal segmental glomerulosclerosis, IgA nephropathy or diabetic kidney disease–an elevated cell senescence rate, determined based on increased expression of the cell-cycle regulator p16^INK4A^ or senescence-associated β-galactosidase, was documented in tubules at the time of diagnosis, and associated with a faster subsequent decline in estimated glomerular filtration rate (Verzola et al., 2020; Esposito et al., 2024). These findings point to accelerated cellular senescence in the tubular compartment as an early event that may contribute to kidney disease progression. In line with this hypothesis, a recent study has identified nicotinamide N-methyltransferase (NNMT) as a key mediator of tubular senescence and kidney fibrosis in early stages of proteinuric chronic kidney diseases, such as diabetic kidney disease, membranous glomerulonephritis and rapidly progressive glomerulonephritis (Chanvillard et al., 2026). This enzyme catalyzes the conversion of nicotinamide and S-adenosyl methionine (SAM) to 1-methylnicotinamide and S-adenosylhomocysteine (SAH), respectively. Analysis of transcriptomic datasets and kidney biopsies from patients with diabetic kidney disease showed increased tubular expression of NNMT, which directly correlated with markers of senescence (Chanvillard et al., 2026). Moreover, in a murine model of early-stage diabetic kidney disease, characterized by glomerular hyperfiltration and moderate albuminuria, the parallel increase in NNMT and senescence markers in the kidney preceded the development of fibrosis (Chanvillard et al., 2026). Further investigation revealed that NNMT overexpression in cultured tubular epithelial cells promoted senescence and pro-fibrotic signaling, whereas its pharmacological blockade by a small-molecule NNMT inhibitor (5-amino-1-methylquinolinium) reversed these effects, likely by restoring metabolic and epigenetic balances. Indeed, the protection provided by NNMT inhibition was associated with an increase in the SAM to SAH ratio in tubular epithelial cells. Since SAM is a universal methyl donor, needed as a co-substrate for all methyltransferases, including DNA and histone methyltransferases, an increase in its availability translated into enhanced methylation and reduced expression of senescence and fibrotic genes. Consistent with the *in vitro* findings, treatment with the small-molecule NNMT inhibitor mitigated fibrotic remodeling in a mouse model of kidney fibrosis (Chanvillard et al., 2026). Collectively, these findings suggest that inhibiting NNMT could be a promising senotherapeutic strategy to protect tubular epithelial cells against maladaptive changes that lead to fibrosis (Table 1).

### 4.2 Responses to proteinuria along the entire nephron

Maladaptive phenotypic and functional responses to proteinuria seem to occur along the entire nephron, as revealed by a study that applied single-nuclei RNA sequencing to kidneys of POD-ATTAC mice (Faivre et al., 2024). In particular, an initial extension of protein reabsorption from segment 1 (S1) to S2 of the proximal tubule was observed in proteinuric mice, which was due to S2 remodeling to a hybrid S1/S2 state (Faivre et al., 2024). Later on, injury and failed-repair cell populations emerged in the proximal tubule. Moreover, the outflow of filtered proteins to the distal tubule led to generalized dedifferentiation of the epithelium, with reduced expression of apical solute transporters (Faivre et al., 2024). The adaptive response of the distal tubule to proteinuria also involves the modulation of ciliary signaling. In rodents and patients with proteinuric chronic kidney diseases, notable elongation of primary cilia was observed in distal tubules, which directly correlated with urinary albumin excretion (Rogg et al., 2025). A combination of *in vitro* and *in vivo* knockout studies suggested that biophysical cues from the glomerular filtrate could inhibit Arp2/3 complex-dependent polymerization of cytoskeletal actin and the subsequent formation of cortical branched actin networks at the apical plasma membrane of tubular cells. These networks are thought to act as physical barriers at the ciliary base that limit the trafficking of cargo to the cilium. Thus, the reduced formation of branched actin networks facilitates cilia formation and elongation in the injured tubule (Rogg et al., 2025). Transcriptome analyses indicate that, following tubular injury, signaling from elongated cilia initially induced a favorable adaptive cell state. However, prolonged ciliary signaling activation eventually contributed to tubular dysfunction through pathological remodeling of the tubular basement membrane (Rogg et al., 2025).

## 5 Inflammation

Several *in vitro* studies have shown that exposure of proximal tubule-derived cells to high concentrations of albumin and other plasma proteins, mimicking the nephrotic milieu, induces NF-κB-dependent expression and secretion of pro-inflammatory chemokines, including monocyte chemoattractant protein-1 (MCP-1), regulated on activation, normal T-cell expressed and secreted (RANTES), IL-8, and fractalkine (Zoja et al., 1998; Morigi et al., 2002; Donadelli et al., 2003; Tang et al., 2003). Proteins internalized into cultured tubular epithelial cells can also lead to the activation of NLRP3 inflammasome, resulting in the maturation of the pro-inflammatory cytokines IL-1β and IL-18 (Fang et al., 2013; Faustino et al., 2018). In *in vivo* models of protein-overload proteinuria these inflammatory substances trigger the recruitment of mononuclear leukocytes, notably T cells and macrophages, into the kidney interstitium (Donadelli et al., 2003; Shimizu et al., 2003). It is increasingly recognized that the functional states of macrophages exist along a continuum, which extends beyond the traditional pro-inflammatory (M1) and anti-inflammatory (M2) dichotomy (Ji et al., 2025). Nonetheless, several studies have identified mediators involved in proteinuria-induced tubulointerstitial recruitment and polarization of macrophages categorized according to the conventional M1/M2 classification. One of such mediators is soluble epoxide hydrolase, an enzyme that converts anti-inflammatory epoxyeicosatrienoic acids to the corresponding, less potent diols (Wang et al., 2018). Its protein levels were found to be higher in proximal tubules from kidney biopsies from patients with IgA nephropathy, with the expression correlating positively with renal macrophage infiltration and the extent of proteinuria (Wang et al., 2018). In *in vitro* experiments, incubation of cultured tubular epithelial cells with purified urine proteins from patients with glomerulonephritis dose-dependently increased soluble epoxide hydrolase expression (Wang et al., 2013, 2018). In turn, the conditioned medium from tubular epithelial cells overexpressing soluble epoxide hydrolase promoted M1 polarization of cultured macrophages through the activation of the NF-κB pathway, whereas soluble epoxide hydrolase inhibition or supplementation with epoxyeicosatrienoic acids fostered M2 polarization through the phosphatidylinositol 3-kinase pathway (Wang et al., 2018). Proximal tubule reabsorption of aberrantly filtered proteins can also promote tubulointerstitial inflammation through the activation of TLR signaling. *In vitro* studies have shown that albumin-stimulated cultured tubular epithelial cells released heat shock protein 70, which promoted the production of pro-inflammatory cytokines *via* a TLR4-dependent pathway (Jheng et al., 2015). Moreover, mice knocked out for *TLR4,* compared to wild-type controls, exhibited less albuminuria, tubular injury and renal macrophage infiltration following streptozotocin-induced diabetes (Jheng et al., 2015). Another study reported that mice with overload albuminuria exhibited activation of Wnt/β-catenin signaling in renal tubules, followed by the production of TLR-4/NLRP3-associated cytokines and chemokines, which orchestrated chemotaxis of pro-inflammatory M1 macrophages into the tubulointerstitium (Wong et al., 2018). In response to overexposure to filtered albumin, tubular epithelial cells can also promote macrophage recruitment and polarization through the secretion of EVs, including exosomes and microvesicles, carrying pro-inflammatory-related cargoes. *In vitro* and 5/6 nephrectomy rat studies have shown that albumin overload triggered tubular epithelial cells to secrete exosomes loaded with CC-chemokine ligand 2 (CCL2, also known as MCP-1) mRNA, which can be internalized by macrophages, leading to their activation and migration (Lv et al., 2018). Similarly, exosomes released from albumin-stimulated tubular epithelial cells and mice with adriamycin-induced proteinuric kidney disease were enriched in miR-19b-3p, which can be taken up by macrophages promoting their M1 polarization by reducing gene and protein expression of suppressor of cytokine signaling-1 (SOCS-1), a negative regulator of the NF-κB signaling pathway (Lv et al., 2020). Other miRNA produced by tubular epithelial cells exposed to albumin can suppress the inflammatory response, such as miR-26a-5p. Blocking exosome secretion from albumin-treated tubular epithelial cells increased intracellular levels of miR-26a-5p, which attenuated inflammation by down-regulating gene expression of the gluthatione-degrading enzyme ChaC glutathione-specific γ-glutamylcyclotransferase 1 (*CHAC1*) and inhibiting NF-κB signaling (Li et al., 2020). IL-4 receptor, which is expressed on proximal tubular epithelial cells, among others, also appears to provide protection against albumin overload-mediated tubulointerstitial inflammation. Following intraperitoneal injection of albumin, mice knocked out for IL-4 receptor α chain experienced more significant tubular injury and kidney infiltration of total macrophages, but with a decrease in M2 phenotype, compared with their wild-type littermates (Peruchetti et al., 2020).

Aberrantly filtered proteins, other than albumin, can also directly promote tubulointerstitial injury. This is the case for isolevuglandin (IsoLG)-modified apolipoprotein A-I (apoAI), which forms under oxidative stress through post-translational modification of the HDL protein apoAI by the reactive lipid aldehyde IsoLG (Zhong et al., 2022). Rodents and patients with proteinuric kidney disease exhibited increased urinary levels of IsoLG-modified apoAI. *In vitro* and *in vivo* studies in a mouse model of proteinuria induced by podocyte-specific injury showed that, compared to unmodified apoAI, IsoLG-modified apoAI is more avidly taken up by tubular epithelial cells, where it promotes injury and inflammation, as evidenced by increased gene expression of KIM-1, NLRP3, IL-1 and IL-6 (Zhong et al., 2022). *In vivo*, inhibition of IsoLG using the reactive aldehyde scavenger pentylpyridoxamine reduced albuminuria, tubular injury and interstitial fibrosis (Table 1) (Zhong et al., 2022). Beyond apoAI, IsoLG can oxidatively modify other proteins to generate adducts that serve as neoantigens and trigger an adaptive immune response in experimental hypertension, which contributes to kidney damage (Kirabo et al., 2014). Under proteinuric and inflammatory states, another antigenic signal may come from albumin-derived peptides generated by the concerted action of proximal tubular cells and dendritic cells, resulting in dendritic cell-induced CD8^+^ T-cell activation (Macconi et al., 2009). An immune response against normally ignored antigens may contribute to chronic kidney disease progression.

## 6 Kidney fibrosis

### 6.1 The role of partial epithelial-to-mesenchymal transition in kidney fibrosis

Kidney fibrosis, considered the final common pathway of proteinuric chronic kidney diseases, is marked by the excessive accumulation of extracellular matrix, eventually leading to scar formation and organ failure. Myofibroblasts, which are characterized by the expression of α-smooth muscle actin (α-SMA), serve as the primary extracellular matrix-producing cells and play a critical role in the fibrotic process (Yamashita and Kramann, 2024). These cells mainly originate from resident fibroblasts and pericytes, with no or minimal contribution from injured epithelial cells (Kuppe et al., 2021). Although they contribute little to the myofibroblast population, injured tubular epithelial cells are thought to actively promote kidney fibrosis through partial epithelial-to-mesenchymal transition (EMT) (Figure 5) (Grande et al., 2015; Lovisa et al., 2015, 2016). During this process, they maintain their epithelial polarity while acquiring the mesenchymal marker α-SMA and the ability to produce pro-fibrotic factors and cytokines, which promote fibroblast-to-myofibroblast differentiation and immune cell recruitment, respectively (Borges et al., 2013; Grande et al., 2015; Lovisa et al., 2015; Youssef and Nieto, 2024). The concept of partial EMT is consistent with findings from an early study using a remnant kidney model showing that, in response to excessive uptake of ultrafiltered proteins, proximal tubular cells increased TGF-β1 transcription and acquired α-SMA expression, with these changes being accompanied by peritubular accumulation of myofibroblasts and inflammatory cells (Abbate et al., 2002).

### 6.2 The role of post-transcriptional and epigenetic mechanisms in kidney fibrosis

Injured tubular epithelial cells can also promote kidney fibrosis through the production of miRNA and long non-coding RNAs. In particular, miR-184 was the most upregulated miRNA in renal tubules of Zucker diabetic fatty rats with overt nephropathy, and associated with interstitial collagen deposition and reduced expression of lipid phosphate phosphatase 3 (LPP3), an enzyme that participates in the catabolism of bioactive lipids involved in multi-organ fibrosis (Zanchi et al., 2017). In cultured proximal tubular cells, albumin was a major trigger for the expression of miR-184, which in turn induced LPP3 downregulation and the upregulation of plasminogen activator inhibitor-1, a serine protease inhibitor that reduces fibrinolysis and incites extracellular matrix accumulation (Zanchi et al., 2017). In Zucker diabetic fatty rats with established kidney disease, lowering albuminuria through treatment with an angiotensin-converting enzyme inhibitor was associated with reduced renal expression of miR-184, preservation of tubular LPP3, and improvement of tubulointerstitial fibrosis (Zanchi et al., 2017). Collectively, these observations point to miR-184 as a downstream effector of albuminuria, which drives kidney fibrosis in rats with diabetic nephropathy (Zanchi et al., 2017). As for long non-coding RNAs, nuclear-enriched abundant transcript 1 (NEAT1) was found to be upregulated in albumin-stimulated cultured tubular epithelial cells and in the kidneys of mice with diabetic kidney disease induced by a high-fat-diet and streptozotocin (Yang et al., 2020). Further gain- and loss-of-function studies *in vitro* and in the above murine model of diabetic kidney disease revealed that Klotho protects against renal tubulointerstitial fibrosis by down-regulating NEAT1, thus reducing NEAT1-mediated expression of pro-fibrotic factors, such as TGF-β1 and connective tissue growth factor, through regulation of the ERK1/2 pathway (Yang et al., 2020). RNA molecules, such as mRNAs and long non-coding RNAs, can undergo various post-transcriptional modifications. Among them, the addition of a methyl group to the nitrogen-6 position of adenosine bases, referred to as N^6^-methyladenosine (m^6^A), has emerged as a key epigenetic mechanism in the pathogenesis of kidney fibrosis. The total m^6^A methylated RNA levels and the expression of methyltransferase-like 3 (METTL3), the main catalytic subunit of the methyltransferase complex responsible for adding methyl groups to RNAs, were found to be higher in the kidneys of mice with unilateral ureteral obstruction and unilateral ischemia-reperfusion injury (Long et al., 2024). In both animal models of kidney fibrosis, proximal tubule-specific knockout of METTL3 reduced kidney accumulation of collagen I and fibronectin, mitigating fibrotic remodeling (Long et al., 2024). Mechanistically, METTL3 enhances m^6^A modification of β-catenin mRNA, which is then stabilized by insulin-like growth factor 2 mRNA-binding protein 3 (IGF2BP3), leading to the expression of its downstream pro-fibrotic genes (Long et al., 2024). The clinical relevance of these findings is highlighted by the substantial expression of METTL3 identified in tubular epithelial cells of kidney biopsies from patients with proteinuric chronic kidney diseases (Jung et al., 2024; Long et al., 2024; Tsai et al., 2024), which directly correlated with the extent of fibrotic lesions (Long et al., 2024; Tsai et al., 2024). Recently, epigenetic suppression of hepatocyte nuclear factor 1 beta (HNF1B), a transcription factor essential for kidney development, has been implicated in chronic kidney disease progression (Isnard et al., 2026). In particular, *HNF1B* inactivation in tubular epithelial cells of adult mice led to tubular atrophy, inflammation and interstitial fibrosis (Isnard et al., 2026). Moreover, in multiple animal models of chronic kidney disease, such as subtotal nephrectomy, Alport syndrome, nephrotic syndrome and ischemia-reperfusion injury, suppression of HNF1B activity was found to precede the appearance of histological lesions (Isnard et al., 2026). The reduced HNF1B activity in injured tubular cells was linked to impaired chromatin accessibility at its binding sites, suggesting epigenetic repression of HNF1B function. Mechanistically, common chronic kidney disease-associated stresses, including albumin exposure and interferon-γ, reduced expression of most HNF1B target genes in tubular cells (Isnard et al., 2026). Together, these data unveiled a self-reinforcing pathological loop whereby loss of HNF1B leads to chronic kidney disease, while chronic kidney disease itself further represses HNF1B activity through epigenetic mechanisms, thereby driving kidney disease progression. The clinical relevance of these findings is underscored by the observation that, in a large cohort of patients with chronic kidney disease, downregulation of HNF1B target genes in kidney biopsies correlated with lower estimated glomerular filtration rate, and with increased interstitial fibrosis and tubular atrophy (Isnard et al., 2026).

### 6.3 The role of metabolic dysfunction in kidney fibrosis

In tubular epithelial cells, in addition to epigenetic regulation of transcription, metabolic dysfunction has also been mechanistically linked to kidney fibrosis. Insulin-like growth factor-binding protein 7 (IGFBP7) plays a critical role in this process. It is a member of the senescence-associated secretory phenotype (Yu et al., 2025b, 2025a) used clinically as a component of the Nephrocheck® urine biomarker test, which predicts the risk of developing moderate to severe acute kidney injury in critically ill, hospitalized patients (Vijayan et al., 2016). In patients with type 2 diabetes, elevated circulating levels of IGFBP7 predicted the onset of major adverse kidney outcomes (Januzzi et al., 2021). Across multiple experimental models of diabetic kidney disease, tubular epithelial cells were identified as the primary source of IGFBP7, which serum and urinary levels directly correlated with albuminuria (Yu et al., 2025a). Loss- and gain-of-function approaches *in vitro* and in murine models of diabetic kidney disease showed that IGFBP7 disrupts mitochondrial bioenergetics in tubular epithelial cells by inhibiting fatty acid oxidation, leading to lipid accumulation, interstitial fibrosis and glomerulosclerosis (Yu et al., 2025a). At the molecular level, IGFBP7 promotes dimerization of acetylated STAT3, which suppresses mitochondrial bioenergetics mediated by AMP-activated protein kinase (AMPK) signaling. Intriguingly, treatment with levomefolic acid, an IGFBP7 inhibitor already used clinically as a nutrient adjuvant, reduced kidney lipid deposition, interstitial fibrosis and glomerular injury in murine models of early and advanced diabetic kidney disease (Table 1) (Yu et al., 2025a).

### 6.4 The role of dysbiosis in kidney fibrosis

In the setting of diabetic kidney disease, dysbiosis has also been implicated in the pathogenesis of renal fibrosis. In this regard, circulating levels of the microbiota-derived peptide corisin were found to be elevated in patients with diabetic kidney disease and in murine models of diabetic kidney fibrosis, where they correlated with kidney dysfunction and increased renal fibrosis, respectively. Molecular dynamic simulations and experimental validation revealed that corisin binds to albumin, which facilitates its transport to the kidney and cubilin-mediated entry into tubular epithelial cells (Yasuma et al., 2025). Further single-cell RNA sequencing analysis suggests that, following cell internalization, corisin induces a senescence-associated secretory phenotype, leading to the secretion of inflammatory cytokines, chemokines and growth factors that promote fibrotic changes (Yasuma et al., 2025). Consistent with these findings, treatment with an anti-corisin monoclonal antibody improved kidney function and attenuated fibrosis in diabetic TGF-β transgenic mice with kidney fibrosis, pointing to corisin as a potential novel therapeutic target (Table 1) (Yasuma et al., 2025).

### 6.5 The role of multi-omics tools in providing novel insights into the mechanisms of kidney fibrosis

A recent study that applied multi-omics tools and spatial transcriptomics to healthy, diabetic and hypertensive diseased human kidneys revealed that cell types other than fibroblasts, myofibroblasts, and injured proximal tubular cells also shape the fibrotic microenvironment (Abedini et al., 2024). These include endothelial cells and immune cells, which follow organizations that resemble early tertiary lymphoid structures and are located near injured proximal tubular cells (Abedini et al., 2024). The cellular and architectural complexity of the fibrotic microenvironment has also been underscored by the intricate interactions among several different cell types, including those between injured proximal tubular cells and fibroblasts, injured proximal tubular cells and immune cells, and fibroblasts and immune cells (Abedini et al., 2024). Interestingly, a gene signature associated with the fibrotic microenvironment predicted kidney disease progression more accurately than traditional histopathologic analysis, suggesting potential clinical value (Abedini et al., 2024).

## 7 Discussion

In recent years, novel insights have been gained regarding the pathophysiology of tubulointerstitial inflammation and fibrosis induced by proteinuria. In this regard, although the proximal tubule is the main target of proteinuria-induced damage, the application of single-nuclei RNA sequencing and other transcriptomic approaches revealed that the detrimental effects of aberrantly filtered proteins involve the whole nephron, in a segment-specific fashion (Faivre et al., 2024; Rogg et al., 2025). Moreover, the combined use of single-cell sequencing and spatial profiling identified dysregulated crosstalk among specific cell populations in fibrotic stroma of human kidneys (Abedini et al., 2024), which could serve as potential therapeutic targets in proteinuric chronic kidney diseases. Epigenetic mechanisms, such as those attributed to increased tubular expression of NNMT (Chanvillard et al., 2026) or METTL3 (Jung et al., 2024; Long et al., 2024; Tsai et al., 2024), have also been involved in the pathogenesis of kidney fibrosis under proteinuric conditions. Despite the promising therapeutic potential of targeting these enzymes, several existing translational barriers remain to be overcome. The most important is achieving effective and specific delivery of NNMT- or METTL3-targeted agents to tubular epithelial cells, in order to avoid off-target effects due to the distribution of these enzymes across multiple tissues and organs. This issue also applies to the therapeutic targeting of the dysregulated expression of non-coding RNAs, such as miRNAs and tRNAs.

In the near future, further mechanistic insights into the toxic effects of ultrafiltered proteins on the tubulointerstitium under proteinuric conditions are expected to emerge from the combined use of multi-omics strategies, such as genomics, transcriptomics, epigenomics, and proteomics. This comprehensive approach will likely facilitate the identification of novel targets for therapeutic intervention. The knowledge that will be gained could also hopefully inform the development of personalized treatment strategies tailored to each patient’s unique biological signature, paving the way for the application of precision medicine to proteinuric chronic kidney diseases.
